# Triage and Diagnostic Accuracy of Online Symptom Checkers: Systematic Review

**DOI:** 10.2196/43803

**Published:** 2023-06-02

**Authors:** Eva Riboli-Sasco, Austen El-Osta, Aos Alaa, Iman Webber, Manisha Karki, Marie Line El Asmar, Katie Purohit, Annabelle Painter, Benedict Hayhoe

**Affiliations:** 1 Self-Care Academic Research Unit (SCARU) Department of Primary Care and Public Health Imperial College London London United Kingdom

**Keywords:** systematic review, digital triage, diagnosis, online symptom checker, safety, accuracy, mobile phone

## Abstract

**Background:**

In the context of a deepening global shortage of health workers and, in particular, the COVID-19 pandemic, there is growing international interest in, and use of, online symptom checkers (OSCs). However, the evidence surrounding the triage and diagnostic accuracy of these tools remains inconclusive.

**Objective:**

This systematic review aimed to summarize the existing peer-reviewed literature evaluating the triage accuracy (directing users to appropriate services based on their presenting symptoms) and diagnostic accuracy of OSCs aimed at lay users for general health concerns.

**Methods:**

Searches were conducted in MEDLINE, Embase, CINAHL, Health Management Information Consortium (HMIC), and Web of Science, as well as the citations of the studies selected for full-text screening. We included peer-reviewed studies published in English between January 1, 2010, and February 16, 2022, with a controlled and quantitative assessment of either or both triage and diagnostic accuracy of OSCs directed at lay users. We excluded tools supporting health care professionals, as well as disease- or specialty-specific OSCs. Screening and data extraction were carried out independently by 2 reviewers for each study. We performed a descriptive narrative synthesis.

**Results:**

A total of 21,296 studies were identified, of which 14 (0.07%) were included. The included studies used clinical vignettes, medical records, or direct input by patients. Of the 14 studies, 6 (43%) reported on triage and diagnostic accuracy, 7 (50%) focused on triage accuracy, and 1 (7%) focused on diagnostic accuracy. These outcomes were assessed based on the diagnostic and triage recommendations attached to the vignette in the case of vignette studies or on those provided by nurses or general practitioners, including through face-to-face and telephone consultations. Both diagnostic accuracy and triage accuracy varied greatly among OSCs. Overall diagnostic accuracy was deemed to be low and was almost always lower than that of the comparator. Similarly, most of the studies (9/13, 69 %) showed suboptimal triage accuracy overall, with a few exceptions (4/13, 31%). The main variables affecting the levels of diagnostic and triage accuracy were the severity and urgency of the condition, the use of artificial intelligence algorithms, and demographic questions. However, the impact of each variable differed across tools and studies, making it difficult to draw any solid conclusions. All included studies had at least one area with unclear risk of bias according to the revised Quality Assessment of Diagnostic Accuracy Studies-2 tool.

**Conclusions:**

Although OSCs have potential to provide accessible and accurate health advice and triage recommendations to users, more research is needed to validate their triage and diagnostic accuracy before widescale adoption in community and health care settings. Future studies should aim to use a common methodology and agreed standard for evaluation to facilitate objective benchmarking and validation.

**Trial Registration:**

PROSPERO CRD42020215210; https://tinyurl.com/3949zw83

## Introduction

### Background

The global shortage of health workers anticipated by the World Health Organization (WHO) is expected to increase from 7.2 million in 2013 to 12.9 million by 2035 [1]. Online symptom checkers (OSCs) have been promoted as a way of supporting more rational use of health care services while saving time for patients, reducing anxiety, and allowing them to take more ownership of their health (self-care) [2,3]. OSCs are web-based tools that can be accessed using a computer, tablet device, or smartphone via a website or an app. On the basis of responses to a series of questions, OSCs may suggest a possible diagnosis and a triage recommendation to inform the next steps [4]. The triage function guides users on whether they should seek a health care assessment, the setting (eg, emergency department [ED] or general practice clinic), and the degree of urgency (eg, immediately, within a few days, or weeks) [5].

The use of OSCs has exploded in recent years. In the United Kingdom, the National Health Service (NHS) 111 online service, which registered 2 million contacts in 2019, reached 7.5 million visits during the first 10 months of 2020, mainly as a consequence of the COVID-19 pandemic [6]. OSCs may indeed provide patients with a more personalized assessment than search engines such as Google [7] and can be used to not only get a diagnosis or a triage recommendation without going to a physician but also learn more about the cause of symptoms or better understand a diagnosis [8]. Studies focused on COVID-19 OSCs showed that these tools tend to have high overall user satisfaction [9] and can help support remote care and self-management, thus reducing the demands on clinicians and health services [10].

However, the potential benefits of OSCs, whether individual or collective, depend primarily on their safety and accuracy. If inadequately designed, they could misdiagnose and misdirect users, potentially diverting them from seeking adequate care or, conversely, placing additional strain on health systems. Two systematic reviews assessed the literature evaluating OSCs [11,12] with mostly weak evidence regarding their diagnostic and triage accuracy. One review focused only on urgent health issues [12], whereas the other included specialty-specific OSCs [11]; both were outdated after the recent publication of several eligible studies.

### Objectives

This systematic review aimed to update and summarize the peer-reviewed literature evaluating the triage accuracy (defined as directing users to appropriate services based on their presenting symptoms) and diagnostic accuracy of OSCs aimed at lay users for general health concerns.

## Methods

This systematic review was conducted following the PRISMA (Preferred Reporting Items for Systematic Reviews and Meta-Analyses) guidelines [13] (Multimedia Appendix 1 [13]).

### Eligibility Criteria

All inclusion and exclusion criteria are presented in Textbox 1.

**Inclusion and exclusion criteria.**

**Inclusion criteria**

- Article type: peer-reviewed articles only
- Language: English
- Publication dates: January 1, 2010, to February 16, 2022
- Population: general population of any age seeking advice digitally regarding how to address, manage, and treat their symptoms and potential health issues, ranging from minor to acute and including long-term conditions
- Intervention: any web-based or digital service that suggests either or both a probable diagnosis and a triage recommendation based on the symptoms inputted by users; these online symptom checkers may be apps, websites, or any other digital platforms (including prototypes) accessible through a mobile phone, tablet device, or computer
- Comparator: triage and diagnosis attached to the vignette or assigned via telephone or face-to-face consultation with a general practitioner or nurse
- Outcomes: quantitative data on diagnostic and triage accuracy of tested online symptom checker
- Study design: observational studies, randomized or nonrandomized controlled trials, controlled before-after studies, or interrupted time series studies

**Exclusion criteria**

- Article type: dissertations, conference proceedings, abstracts, and all non–peer-reviewed papers
- Language: any language other than English
- Publication dates: before 2010 or after February 16, 2022
- Population: specific age or patient group (eg, patients with COVID-19 symptoms or children only)
- Intervention: tools that only provide an asynchronous web-based consultation (eg, via email) or health advice without diagnosis or triage, as well as those that were specific to age, disease, or specialty
- Comparator: no comparator
- Outcomes: not applicable
- Study design: all study designs other than observational studies, randomized or nonrandomized controlled trials, controlled before-after studies, or interrupted time series studies

### Search Strategy

A scoping review was conducted after consulting with a research librarian to help establish search terms. An initial list of search terms was compiled and applied to MEDLINE and Embase to confirm the relevance of the results. Reference lists from several relevant studies and similar reviews were manually searched to expand the search terms and refine the search strategies. Medical Subject Headings were adapted for each database. Searches were carried out on February 17, 2022 (searching for studies published between January 1, 2010, and February 16, 2022). We searched the following 5 databases: MEDLINE, Embase, CINAHL, Health Management Information Consortium (HMIC), and Web of Science. No manual searching was performed, but we screened the references of all studies selected for full-text screening. The final list of search terms for each database is presented in Multimedia Appendix 2.

### Study Selection

The studies retrieved were first imported into EndNote X7 (Clarivate) to help identify and remove duplicates. The included studies were then entered in Covidence (Veritas Health Innovation Ltd), where additional duplicates were removed. Titles and abstracts were screened by 2 researchers. The full text of potentially eligible studies was then independently assessed by 2 researchers. Studies where the primary reviewers disagreed were reviewed independently by a third researcher; any remaining disagreement was resolved through team discussion.

### Data Extraction

After full-text screening, data extraction was carried out by 2 researchers independently for each study using a comprehensive standardized extraction form designed for the specific characteristics of this review and refined after the testing of 2 (14%) of the 14 studies. Key areas of data collection were the study sample size and characteristics; reference standard, measures, and levels of triage and diagnostic accuracy; and any additional comparator and reported outcomes. The detailed data extraction table is presented in Multimedia Appendix 3 [14-27].

### Risk of Bias and Applicability

Two researchers independently assessed the risk of bias and applicability concerns using a revised version of the Quality Assessment of Diagnostic Accuracy Studies-2 (QUADAS-2) tool [28] for the domains of patient selection, performance of the index test, performance of the reference test, and flow and timing (for risk of bias only). Conflicts were resolved through discussion. No study was excluded based on quality assessment. We also assessed the overall strength of evidence (quality and relevance) for both main outcomes using an adaptation of the method described by Chambers et al [12] in their review. This involved classifying evidence based on study numbers, risk of bias, and levels of consistency among the findings.

### Analysis

We performed a descriptive narrative synthesis as well as a strength-of-evidence assessment structured around the prespecified research questions and outcomes to describe the collective findings of the included studies. Wide variations in design and methodology made meta-analyses impractical.

## Results

**Figure 1:**
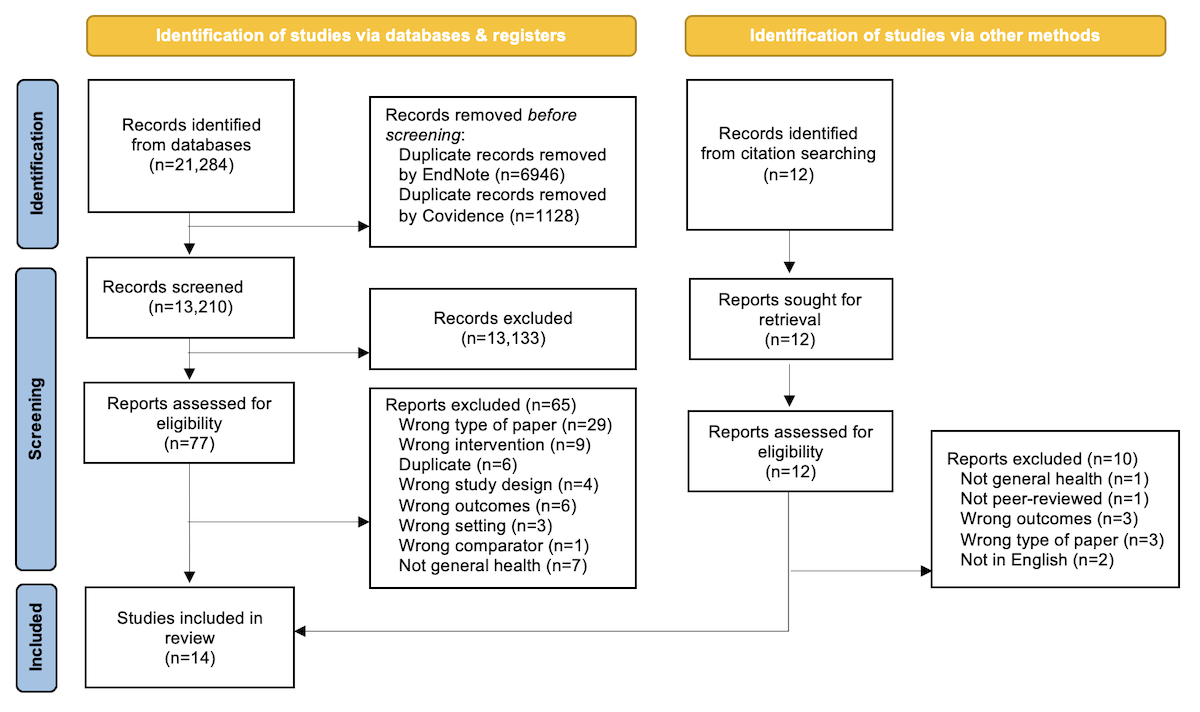
PRISMA (Preferred Reporting Items for Systematic Reviews and Meta-Analyses) flow diagram.

A total of 21,284 records were identified through initial searches, with an additional 12 studies identified through citation searching. Of the 21,296 studies, 14 (0.07%) were included in the review (Figure 1).

### Characteristics of the Included Studies

Table 1 shows the main characteristics of the included studies published between 2014 and 2022. Of the 14 studies, 5 (36%) were conducted with participants based in the United States [14-18], three (21%) in the United Kingdom [19-21], two (14%) in Australia [22,23], two (14%) in Canada [24,25], one (7%) in the Netherlands [26], and one (7%) in Hong Kong [27]. Seven (50%) of the 14 studies [14,15,17,18,21-23] used standardized patient vignettes; several (3/7, 43%) were inspired by, or included, the 45 vignettes used by Semigran et al [14,15]. The remaining studies (7/14, 50%) used data from real patients through either their medical health records [16,27] or direct input by users [19,20,24-26] in different settings, including primary care and emergent care settings. Population sizes ranged from 45 to 25,333 patients.

Of the 14 studies, 7 (50%) evaluated a single OSC [17-20,24-26], whereas the other 7 (50%) tested and compared the performance of two [27] to thirty-six [22] OSCs. Where provided, the most common justifications for selection were language (English), the level of popularity among users, and accessibility (free). The most frequently included OSC was WebMD (included in 6/14, 43% studies), followed by Isabel and Symptomate (tested in 5/14, 36% studies) and Drugs.com, Symcat, and FamilyDoctor (each tested in 4/14, 29% studies). The complete list of tested OSCs is presented in Multimedia Appendix 4 [14-27], along with measurements used to assess their diagnostic and triage accuracy.

**Table 1:** Main characteristics of the included studies.

Authors, year; country	Study design	OSCs^a^, n	Population or sample	Reference standard	Additional comparator
Poote et al [19], 2014; United Kingdom	Prospective cohort study	1	A total of 154 patients from a PC^b^ student health center; age: 17 to 43 years (mean age: 22 years); 64.3% female and 35.7% male	Seven GPs^c^ through F2F^d^ consultation	N/A^e^
Semigran et al [14], 2015; United States	Vignette cohort study	23	A total of 45 standardized patient vignettes; mean age 34.02 (SD 22.48) years; age range 4 months to 77 years; 38% female and 62% male	Diagnostic and triage recommendations attributed to the vignettes	N/A
Semigran et al [15], 2016; United States	Vignette cohort study	23	Same as Semigran et al [14]	Diagnostic recommendations attributed to the vignettes	A total of 234 GPs through the Human Dx^f^ platform
Verzantvoort et al [26], 2018; the Netherlands	Prospective, cross-sectional cohort study	1	A total of 126 app users; 52% female and 48% male	Telephone triage by a nurse	N/A
Berry et al [16], 2019; United States	Retrospective cohort study	5	A total of 168 ED^g^ patient records with prior diagnosis of HIV or hepatitis C; mean age 44.9 (SD 12.3) years; 36.9% female and 63.1% male; 38% Black and 62% White	ED physician through F2F consultation and triage all deemed emergent as patients presented to the ED	N/A
Gilbert et al [17], 2020; United States	Vignette cohort study	8	A total of 200 standardized patient vignettes; mean age 35.59 (SD 24.48) years; age range 1 month to 89 years; 57% female and 43% male	Diagnostic and triage recommendations attributed to the vignettes	A total of 7 GPs through telephone consultation; gold standard set by 2 panels of 3 GPs each for diagnosis and triage
Hill et al [22], 2020; Australia	Vignette cohort study	36	A total of 48 standardized patient vignettes (including 30 adapted from Semigran et al [14]); mean age 21.2 (SD 10.2) years; age range 4 weeks to 77 years; 43.75% female and 56.25% male	Diagnostic and triage recommendations attributed to the vignettes and confirmed by 2 GPs and 1 ED specialist	N/A
Yu et al [27], 2020; Hong Kong	Retrospective cohort study	2	A total of 149 real A&E^h^ patient charts; Drugs.com: mean age 55.6 years; 58% female and 42% male; FamilyDoctor: mean age 55.4 years; 55% female and 45% male	Triage categories assigned by the triage nurses using A&E department triage protocols	N/A
Ceney et al [21], 2021; United Kingdom	Vignette cohort study	12	A total of 50 standardized patient vignettes (including 44 from Semigran et al [14] and an additional 6 to account for depression or COVID-19 infection)	Diagnostic recommendations attributed to the vignettes and triage recommendations according to NICE^i^ guidance	N/A
Chan et al [25], 2021; Canada	Prospective cohort study	1	A total of 581 patients (281 ED patients and 300 PC patients); ED patients: mean age 38 (SD 16; range 16-91) years; PC patients: mean age 48 (SD 18; range 16-91) years; 63% female and 37% male	Triage by GP through F2F consultation and reviewed by 2 physician authors who, by consensus, assigned a corresponding triage recommendation	N/A
Delshad et al [18], 2021; United States	Vignette cohort study	1	A total of 50 standardized patient vignettes; mean age 47.1 (SD 19.8) years; age range 20 to 84 years; 50% female and 50% male	Three consensus triage recommendations attributed to the vignettes by 14 GPs	Triage decisions by 14 individual GPs
Gilbert et al [23], 2021; Australia	Vignette cohort study	1	Same as Hill et al [22]	Triage recommendations set in Hill et al [22], and 1 clinician (with GP and ED experience) decided whether the diagnostic recommendations provided by the app matched the one set by Hill et al [22]	N/A
Trivedi et al [24], 2021; Canada	Prospective observational study	1	A total of 429 patients; mean age 47 (SD 22) years; 50.2% female and 49.8% male	CTAS^j^ scores assigned face to face by the dedicated ED triage nurse	N/A
Dickson et al [20], 2022; United Kingdom	Retrospective cohort study	1	A total of 25,333 patients; median age 46 (range 30-62) years; 54.2% female and 45.8% male	MTS^k^ triage categories assigned face to face by a triage nurse	N/A

^a^OSC: online symptom checker.

^b^PC: primary care.

^c^GP: general practitioner.

^d^F2F: face-to-face.

^e^N/A: not applicable.

^f^Human Dx: Human Diagnosis Project.

^g^ED: emergency department.

^h^A&E: Accident & Emergency.

^i^NICE: National Institute for Health and Care Excellence.

^j^CTAS: Canadian Triage and Acuity Scale.

^k^MTS: Manchester Triage System.

### Diagnostic Accuracy

The diagnostic accuracy of the tested OSC was reported in 50% (7/14) of the included studies [14-17,21-23]. Significant variability in the levels of diagnostic accuracy of OSCs was observed among individual OSCs and studies, but the diagnostic accuracy was deemed to be low overall and, on average, lower than that of general practitioners (GPs) when compared [15,17]. Table 2 presents the levels and ranges of average diagnostic accuracy, defined as listing the correct diagnosis first, as well as the main variables assessed by each study.

There was agreement regarding the general impact of condition frequency with a better average diagnostic accuracy observed for *common* conditions than for *uncommon* conditions in 2 (14%) of the 14 studies [14,22], but the findings were conflicting regarding the influence of condition urgency on diagnostic accuracy [14,21,22]. Hill et al [22] also found that the 8 OSCs using artificial intelligence (AI) algorithms had a better diagnostic accuracy overall: they listed the correct diagnosis first for 46% (95% CI 40%-57%) of the vignettes compared with only 32% (95% CI 26%-38%) for the 19 other tested OSCs. However, these authors noted that “information about whether programs employed AI algorithms was drawn solely from that provided in the [O]SC,” which is problematic because definitions of AI and algorithms may vary among studies and OSCs, with some authors restricting AI to machine learning methods, whereas others included Bayesian methods or even simple rules-based algorithms. Finally, the source of the OSC, namely the Apple App Store or Google Play Store, was found to affect diagnostic accuracy in 1 (7%) of the 14 studies [22].

**Table 2:** Levels of average diagnostic accuracy (ADA) and main variables identified.

Authors, year; country	OSCs^a^, n	OSC ADA (listing the correct diagnosis first)			ADA range, % (OSC) to % (OSC)		Main variables identified		ADA of additional comparator	
		Values, % (95% CI)	Values, mean (SD)					Values, % (95% CI)		Values, mean (SD)
Semigran et al [14], 2015; United States	23	34 (31-37)	—^b^	5 (MEDoctor) to 50 (DocResponse)		Urgency ↓^c^ Frequency ↑^d^ Demographic data ↔^e^ Maximum number of diagnoses provided ↔ Distributor ↔ Nurse triage protocol ↔		N/A^f^		N/A
Semigran et al [15], 2016; United States	23	34 (31-37)	—	5 (MEDoctor) to 50 (DocResponse)		None		72.1 (69.5-74.8)^g^		—
Berry et al [16], 2019; United States	5	—	—	3 (WebMD) to 16.4 (Symcat)		None		N/A		N/A
Gilbert et al [17], 2020; United States	8	—	26.1 (8.9)	18 (Symptomate) to 48 (Ada)		NHS^h^ vignettes (based on transcripts of real calls made to NHS Direct) ↓		—		71.2 (5.6)^i^
Hill et al [22], 2020; Australia	36	36 (31-42)	—	12 (ePain Assist) to 61 (Symptomate)		Urgency ↑↓^j^ Frequency ↑ AI^k^ ↑ Demographic data ↑ Maximum number of diagnoses provided ↔ Apple vs Google ↑↓		N/A		N/A
Ceney et al [21], 2021; United Kingdom	9^l^	37.7 (33.6-41.7)	—	22.2 (Caidr) to 72 (Ada)		Urgency ↓ Number of questions ↑ Time to complete ↑		N/A		N/A
Gilbert et al [23], 2021; Australia	1^m^	65^n^	—	N/A		Australian-specific vignettes ↓		N/A		N/A

^a^OSC: online symptom checker.

^b^Not stated.

^c^increases average diagnostic accuracy.

^d^↑: decreases average diagnostic accuracy.

^e^↓: no substantial influence on average diagnostic accuracy.

^f^↔: N/A: not applicable.

^g^A total of 234 general practitioners on the Human Diagnosis Project platform.

^h^NHS: National Health Service.

^i^Seven GPs through telephone consultation.

^j^↑↓: mixed impact on average diagnostic accuracy.

^k^AI: artificial intelligence.

^l^Out of 12 online symptom checkers.

^m^Ada.

^n^95% CI values not provided.

### Triage Accuracy

With the exception of the study by Semigran et al [15], all studies reported on the selected OSCs’ triage accuracy, which seemed to be suboptimal overall. Levels of average triage accuracy are presented in Table 3. A triage was deemed accurate only when it matched the one attributed by ≥1 clinicians as the *gold standard*. In the study by Berry et al [16], however, all cases were “expected to be mostly emergency” because they were records of patients presenting to the ED. This was surprising because triage advice, that is, whether and where users should seek a health care assessment for their presenting symptoms, is precisely one of the main functions of OSCs, with several studies showing that laypersons tend to be biased toward overtriage, while also missing emergency cases [29-31]. In addition, as Chan et al [25] pointed out in their review, and as others have shown [32], if patients decide to present to the ED, it does not mean that they automatically qualify for emergency treatment, thus undermining the pertinence of the findings of Berry et al [16] regarding triage accuracy.

Triage accuracy seemed to be affected by the level of urgency of the condition as shown in 5 (36%) of the 14 studies [14,21,22,24,27]. Of these 5 studies, 3 (60%) found that triage accuracy increased with the urgency of the condition [14,21,22]. The results regarding the frequency of the condition were more conflicting, depending on the studies and OSCs. According to Hill et at [22], the accuracy of the 5 OSCs requiring demographic data (defined as requesting “at least age and sex”) was on average greater than that of the OSCs in the 14 studies that did not require demographic data. In the study by Semigran et al [14], OSCs that used the Schmitt or Thompson nurse triage protocols were more likely to provide appropriate triage decisions. Finally, 2 (15%) studies [14,22] found that some of the OSCs (including iTriage, Symcat, Everyday Health, Doctor Diagnose, Symptomate, and Isabel) never recommended *self-care* and therefore could not match this triage category.

Specific characteristics of the study population may also affect the levels of triage accuracy of OSCs. Berry et al [16] found that a significantly higher percentage of patients with hepatitis C virus infection received a “correct diagnosis” than patients with HIV infection, both remaining low, however, leading the authors to conclude that current OSC software algorithms may not account for patient populations with complex, immunocompromised HIV infection and hepatitis C virus infection. Only 2 (14%) of the 14 studies [19,24] looked at the impact of users’ age and gender [19] or age and sex [24] on triage accuracy and found diverging results. Finally, methodological choices relating to the type or source of the vignettes also affected diagnostic accuracy (eg, vignettes made up by researchers vs vignettes based on transcripts of real calls made to NHS Direct [17] or Australian-specific vignettes [23]).

**Table 3:** Levels of average triage accuracy (ATA) and main variables identified.

Authors, year; country	OSCs^a^, n	OSC ATA		ATA range, % (OSC) to % (OSC)	Main variables identified	ATA of additional comparator	
		Values, % (95% CI)	Values, mean (SD)			Values, % (95% CI)	Values, mean (SD)
Poote et al [19], 2014; United Kingdom	1	39^b^	—^c^	N/A^d^	Age ↔^e^ Gender ↔	N/A	N/A
Semigran et al [14], 2015; United States	15^f^	57 (52-61)	—	33 (iTriage) to 78 (HMS Family Health Guide)	Urgency ↑^g^ Frequency ↓^h^ Schmitt or Thompson nurse triage protocols ↑	N/A	N/A
Verzantvoort et al [26], 2018; the Netherlands	1	81^b^	—	N/A	None	N/A	N/A
Berry et al [16], 2019; United States	5	45.8^b^	—	—	More patients with hepatitis C virus infection received a correct triage than patients with HIV infection	N/A	N/A
Gilbert et al [17], 2020; United States	8	—	90.1 (7.4)	80 (Buoy) to 97.8 (Symptomate)	NHS^i^ vignettes (based on transcripts of real calls made to NHS Direct) ↓	N/A	97.0 (2.5)^j^
Hill et al [22], 2020; Australia	19^k^	49 (44-54)	—	17 (Doctor Diagnose) to 61 (Healthdirect)	Urgency ↑ Frequency ↑ Demographic data ↑ AI^l^ algorithm ↔ Maximum number of diagnoses provided ↔	N/A	N/A
Yu et al [27], 2020; Hong Kong	2	62^b^	—	50 (FamilyDoctor) to 74 (Drugs.com)	Urgency ↑	N/A	N/A
Ceney et al [21], 2021; United Kingdom	10^m^	57.7 (53.2-62.2)	—	35.6 (Caidr) to 90 (Doctorlink)	Urgency ↑ Number of questions ↔	N/A	N/A
Chan et al [25], 2021; Canada	1	73^b^	—	N/A	None	58^b,n^	N/A
Delshad et al [18], 2021; United States]	1	Consensus A: 85^b^, consensus B: 92^b^, and consensus C: 88^b^	—	N/A	None	Consensus A: 82^b^, consensus B: 69^b^, and consensus C: 80^b,o^	N/A
Gilbert et al [23], 2021; Australia	1	63^b^	—	N/A	Australian-specific vignettes ↓	N/A	N/A
Trivedi et al [24], 2021; Canada	1	27^b^	—	N/A	Urgency ↑↓^p^ Sex: More female patients received a correct triage than male patients Age: more patients in the 20 to 39 years age group received a correct triage than other age groups Cardiorespiratory problems ↑	N/A	N/A
Dickson et al [20], 2022; United Kingdom	1	30.7^b^	—	N/A	None	N/A	N/A

^a^OSC: online symptom checker.

^b^95% CI values not provided.

^c^Not stated.

^d^N/A: not applicable.

^e^↔: no substantial influence on average triage accuracy.

^f^Out of 23 online symptom checkers.

^g^↑: increases average triage accuracy.

^h^↓: decreases average triage accuracy.

^i^NHS: National Health Service.

^j^Seven general practitioners through telephone consultation.

^k^Out of 36 online symptom checkers.

^l^AI: artificial intelligence.

^m^Out of 12 online symptom checkers.

^n^Patients’ decision.

^o^Triage by 14 individual general practitioners.

^p^↑↓: mixed impact on average triage accuracy.

### Additional Reported Outcomes

Of the 14 studies, 9 (64%) assessed under- and overtriage by OSCs [14,17,19,21,22,24-27]. Of these 9 studies, 5 (56%) found that OSCs tend to overtriage (ie, be risk averse) [14,19,24-26], which is defined as encouraging users to seek care in a setting or with a degree of urgency that is not strictly necessary for the presenting symptoms. Overtriage is likely due to concerns about patient safety and product liability. However, most of the studies (5/9, 56%) observed that undertriage did occur. Yu et al [27] found that Drugs.com and FamilyDoctor undertriaged 24% (95% CI 16%-34%) and 45% (95% CI 35%-55%) of the cases, respectively. Chan et al [25] estimated that compliance with the triage recommendations in their cohort could have reduced hospital visits by 55% but would also cause potential harm from delayed care in 2% to 3% of the cases. Ceney et al [21] found that all 12 OSCs tested led to additional resource use, ranging between 12.5% (95% CI 6.1%-33.5%) for the OSC with the lowest impact and 87.5% (95% CI 52.8%-100%) for the OSC with the highest impact. It is pertinent that such estimates are based on the assumption that users follow the advice provided by the OSC, which none of the included studies assessed. However, Verzantvoort et al [26] did report that 65% of the users intended to follow the OSC tool advice. Gilbert et al [17] reported on the coverage, comprehensiveness, and relevance of each OSC. Furthermore, Dickson et al [20] reported that the median time for nurse triage was 17 (IQR 9-31) minutes compared with the median time of 5 (IQR 4-6) minutes for eTriage.

### Risk of Bias Within Studies

The evaluation of the risk of bias and applicability was conducted using the revised QUADAS-2 tool, and the results are summarized in Table 4. This assessment revealed that all studies had at least 1 area with unclear risk of bias, and 6 (43%) of the 14 studies had a high risk of bias; for instance, Yu et al [27] replaced cases with chief complaints not available on the OSCs with more compatible ones, which, according to the authors, likely resulted in overestimated accuracy levels of the OSCs. Dickson et al [20] acknowledged the possibility of selection bias owing to the perceptions of reception staff regarding the ability of older patients to use the OSC, which resulted in its reduced use by patients aged >70 years. In the study by Poote et al [19], the GP assessing the patients’ conditions had access to the index test results, which means that the reference standard was not blinded to the index test results. In the study by Hill et al [22], the lack of data regarding the blinding of the inputters to the diagnostic or triage recommendations, as well as their familiarity with the system, introduced a risk of bias regarding the conduct of the index test. The affiliation of authors is another source of bias because several of the included studies were conducted by authors working for OSC developers; for example, in the study by Gilbert et al [23], 4 of the 5 authors worked for the tested app, Ada.

**Table 4:** Risk-of-bias summary using the revised version of the Quality Assessment of Diagnostic Accuracy Studies-2 (QUADAS-2) tool.

Study	Risk of bias					Applicability concerns		
	Patient selection	Index test	Reference standard	Flow and timing	Patient selection		Index test	Reference standard
Poote et al [19]	+^a^	+	−^b^	?^c^	?		+	?
Semigran et al [14]	+	+	?	+	?		+	?
Semigran et al [15]	?	+	?	?	?		+	+
Verzantvoort et al [26]	?	+	?	−	+		+	+
Berry et al [16]^d^	+	?	−	?	+		?	+
Gilbert et al [17]	+	?	+	+	+		?	+
Hill et al [22]	+	−	+	?	+		?	+
Yu et al [27]	−	+	?	?	+		+	+
Ceney et al [21]	+	+	+	?	+		+	+
Chan et al [25]	?	+	+	?	+		+	+
Delshad et al [18]	?	?	+	?	+		+	+
Gilbert et al [23]	+	?	?	+	+		+	+
Trivedi et al [24]	?	?	+	?	?		?	+
Dickson et al [20]	−	+	+	?	?		+	+

^a^+: low risk of bias.

^b^−: high risk of bias.

^c^?: unclear risk of bias.

^d^In this study, the reference standard for the triage accuracy and the reference standard for the diagnostic accuracy were different. Only the one for the triage accuracy had a high risk of bias.

### Overall Strength-of-Evidence Assessment

The overall strength of evidence for key outcomes is summarized in Table 5. Although there is rather strong evidence that the diagnostic accuracy of OSCs tends to be lower than that of health care professionals (HCPs), the strength of evidence is more variable regarding triage accuracy.

**Table 5:** Overall strength of evidence by main outcome.

Outcome and reference standard (and additional comparator)		Relevant studies	Evidence statement	Strength of evidence
**Diagnostic accuracy**				
	GP^a^ (F2F^b^ consultation)	Berry et al [16]^c^	Despite great variations among OSCs^d^, overall diagnostic accuracy was deemed to be low and always lower than that of the reference standard.	Moderate
	Attributed to the vignette	Semigran et al [14]^c^ Ceney et al [21]^c^	Despite great variations among OSCs, overall diagnostic accuracy was deemed to be low and always lower than that of the reference standard.	Strong
	Attributed to the vignette and confirmed by GPs	Hill et al [22]^c^ Gilbert et al [23]^c^	Despite great variations among OSCs, overall diagnostic accuracy was deemed to be low and always lower than that of the reference standard.	Strong
	Attributed to the vignette GPs as additional comparator	Gilbert et al [17]^c^ Semigran et al [15]^c^	Despite great variations among OSCs, overall diagnostic accuracy was deemed to be low and always lower than that of the reference standard and of the comparator (GPs).	Strong
**Triage accuracy**				
	Triage nurses	Verzantvoort et al [26]^c^ Trivedi et al [24]^c^ Dickson et al [20]^c^ Yu et al [27]^c^	Despite some variations among OSCs, including relatively high levels of triage accuracy in the study by Verzantvoort et al [26], triage accuracy was always lower than that of the reference standard. However, the study by Verzantvoort et al [26] and the study by Yu et al [27] both had 1 area each with a high risk of bias.	Moderate
	GPs	Poote et al [19]^c^ Delshad et al [18]^e^	Findings were inconsistent, with great variations between studies that evaluated 2 different OSCs. However, the reference standard chosen in the study by Poote et al [19]^c^ has a high risk of bias.	Inconsistent
	GPs Patients’ self-triage as additional comparator	Chan et al [25]^e^	Overall triage accuracy was lower than that of the reference standard but considered high and higher than that of the additional comparator (patients).	Moderate
	Attributed to the vignette	Semigran et al [14]^c^ Gilbert et al [23]^c^	There was great variation among OSCs. Overall triage accuracy was always lower than that of the reference standard and considered to be low.	Strong
	Attributed to the vignette & confirmed by GPs	Hill et al [22]^c^	Despite some variations among OSCs, triage accuracy was deemed to be low and always lower than that of the reference standard. However, the index test chosen has a high risk of bias.	Weak
	Attributed to the vignette GPs as additional comparator	Gilbert et al [17]^f^	There was great variation among OSCs. Overall triage accuracy was always lower than the reference standard, but some OSCs performed almost as well as the additional comparator (GPs).	Moderate
	Patients’ self-triage	Berry et al [16]^c^	Overall triage accuracy was deemed to be low and always lower than reference standard. However, the reference standard chosen has a high risk of bias.	Weak
	NICE^g^ guidance	Ceney et al [21]^c^	Overall triage accuracy was deemed to be low, with a few exceptions and great variations among OSCs but always lower than the reference standard.	Moderate

^a^GP: general practitioner.

^b^F2F: face-to-face.

^c^Worst outcome with online symptom checkers.

^d^OSC: online symptom checker.

^e^Better outcome with online symptom checkers.

^f^Varying results within study.

^g^NICE: National Institute for Health and Care Excellence.

## Discussion

### Principal Findings

Evidence on the triage and diagnostic accuracy of OSCs suggests that they are currently not a viable replacement for other triage and diagnostic options such as telephone triage or in-person consultations. Furthermore, some of the OSCs performed well regarding triage accuracy but poorly regarding diagnostic accuracy and vice versa. Studies evaluating various OSCs also revealed important performance variations among them. Several of the studies (5/14, 36%) found that the condition’s frequency (2/14, 14%) and urgency (5/14, 36%) could affect diagnostic and triage accuracy levels but with mixed conclusions. In addition, some specific OSC characteristics may also play a role, including the use of AI, self-reported demographic and anthropomorphic data, the maximum number of diagnoses provided, or the use of nurse triage protocols. Some characteristics of the *study population* were also shown to affect the level of triage and diagnostic accuracy, including the source of the vignettes as well as the health status of patients or the geographic specificity of diseases and symptoms. The safety of the triage recommendation as well as the tendency to over- or undertriage were important outcomes associated with triage accuracy. These also resulted in some of the studies (3/13, 23%) estimating the potential impact on service use, which diverged among studies, partly because some of the tools promoted overuse of services whereas others tended to undertriage users.

### Strengths and Limitations

We conducted a comprehensive search by repeatedly revising and reviewing our search strategy and search terms, including manually searching reference lists. Highly inclusive searches yielded a significant number of initial results, which we screened in pairs to limit errors. However, we acknowledge that eligible studies might have been excluded or omitted and that relevant papers in gray literature or papers written in languages other than English or published before 2010 might also have been excluded owing to our selection criteria. The included studies were all conducted in high-income countries, which may limit the wider generalizability of the findings. Comparison among studies was particularly difficult because of the variety of study designs, outcome measures, populations, and tools considered. In addition, 4 (29%) of the 14 studies evaluated >10 OSCs, adding to the complexity of comparisons. Triage accuracy, which consistently appeared as the main outcome of interest across studies, was measured using varying numbers of categories as well as different time periods and triage locations, thus limiting further the possibility for objective head-to-head comparisons. The lack of a common methodology for evaluating OSCs strongly limits the possibility of comparison among tools and studies. It is pertinent too that all 14 studies had at least 1 area with an unclear risk of bias, and 6 (43%) of the 14 studies had a high risk of bias.

### Comparison With Prior Work

Two previous systematic reviews assessed the literature on a similar topic. The 2019 systematic review by Chambers et al [12] included any type of publication, including gray literature, but was limited to studies relating to urgent health issues only. The evidence was assessed as being mostly weak and insufficient to determine the level of safety of digital symptom checkers and OSCs for patients. More recently, Wallace et al [11] published a systematic review on the diagnostic and triage accuracy of OSCs, including specialty-specific tools, but the search was restricted to MEDLINE and Web of Science up to February 15, 2021. Both triage accuracy and diagnostic accuracy of the OSCs were found to be mostly low despite variations. Reliance on these tools was therefore considered as posing a potential clinical risk. The identification of 7 new studies published since mid-February 2021, along with increasing use of OSCs during the COVID-19 pandemic despite cautionary calls, motivated us to conduct this review.

This review aimed to not only update but also strengthen the quality of the evidence by including only peer-reviewed papers and focusing on OSCs for general health concerns (nonspecialty specific). However, the evidence remains inconsistent and calls both for caution in promoting OSCs and the need for further studies to improve and inform future development of these tools.

### Implications for Research and Practice

Most of the included studies (10/14, 71%) highlighted that OCS performance tended to remain low and that further improvements, testing, and research are needed. Although there is a sense in commentaries and previous studies [12] that OSCs tend to overtriage and thus should be considered *risk averse*, our review identified several instances of *undertriage* among OSCs. This finding is concerning because it suggests a risk of delay in accessing care for individuals using these decision-support tools. The impact of overtriage on health services must also be considered because this might negatively affect the quality of services provided and thus ultimately represent a risk for service users. Additional work is urgently needed to understand the extent and implications of inappropriate triage recommendations of existing publicly available OSCs, which require a real-life assessment of rates of user compliance with the tool’s advice.

The ability of OSCs to change or direct the behavior of users through the provision of triage recommendations remains unclear; this has significant implications for their likely impact on health service use and health outcomes. Current evidence on user compliance remains scarce and inconsistent: studies with relatively positive results tend to be limited to users’ intention to follow the recommendation [26,33] or to experiments with vignettes instead of users’ own symptoms [34]. Meanwhile, 2 other studies reported less encouraging results, with few users following the ED visit advice [8] or a majority of patients in a primary care waiting room not changing their decision to see the GP despite the OSC’s alternative advice to wait and self-care [35]. Interestingly, the evaluation of the telephone advice and triage service NHS 111 also showed poor compliance with advice, with 11% of the patients advised to self-care or seek primary care attending the ED [36].

Four (29%) of the included 14 studies offered suggestions for improvement of OSCs, including incorporating local, regional, and seasonal epidemiological data, along with individual clinical data [14,27], as well as more efficient inclusion of demographic data in the algorithm [14]. Authors also suggested that, despite their limitations, OSCs could still be useful for tracking epidemiological data, self-education of users about their health, improving patient-physician relationships and directing users to appropriate care (especially for tools that are directly linked with health care services) [22], and supporting the use of AI-based symptom assessment technology in diagnostic decision support for GPs [17].

More studies are needed to clearly assess the triage and diagnostic accuracy of OSCs for all potential users. The lack of consensus on how OSCs should be evaluated by any national or international regulatory body means that developers produce their own evidence to validate products to meet regulatory requirements (UK Conformity Assessed [UKCA] and Conformité Européenne [CE] markings). There is a need for additional research into the methods of evaluating OSCs, including how to establish a gold standard response and determine appropriate accuracy and safety scores in comparison with this gold standard. A consensus agreement on what could be deemed an *acceptable* rate of under- or overtriage would also be required. Specific evidence standards should be provided for OSCs to augment existing guidance, such as the National Institute for Health and Care Excellence (NICE) evidence standards framework and the evaluation requirements for medical device certification with the Medicines & Healthcare Products Regulatory Agency (MHRA). A set of congruent requirements for the standardized vignette-based clinical evaluation process of OSCs has been proposed with this aim [37].

Future studies should ideally be based on the direct input of real-life patients, who would be best placed to enter their own symptoms into the OSCs to allow a better assessment of real-world performances, instead of mostly fictional clinician-authored vignettes or medical records drafted and entered by researchers who are likely to be prone to bias. In addition, the study populations should be broad and diverse in terms of race, age, sex, gender, social class, education, and abilities because these characteristics have been correlated with differential and possibly discriminatory treatment by an HCP in real-life encounters in multiple countries and settings [38-41]. For several communities and individuals, including ethnic minorities, migrants, and women, as well as gender nonconforming and lesbian, gay, bisexual, transgender, and queer (LGBTQ) communities [42], the use of an OSC might potentially represent a safer, more accessible, and more accurate option than a real-life encounter with an HCP. However, if these communities are not included and accounted for in the design and testing of digital technology, including OSCs, such discriminations might be further reinforced [43]. Achieving health equity requires a shift in methodologies and perspectives, including the adoption of a feminist intersectional lens in digital health [44]. Finally, although OSCs may be perceived as useful [8], there may also be issues in understanding and interpreting the recommendations provided [45], making accessibility, usability, and interpretability the key factors to consider when designing, promoting, and evaluating these tools.

In response to the limitations inherent in current evaluations of OSCs, several authors have called for a multistage-process evaluation of increasing exposure to real-life clinical environments in proportion to OSC system maturity, taking place both before and after the tool’s launch and including the testing of different aspects of the OSC, such as usability, effectiveness, and safety [46-51].

### Conclusions

OSCs have a significant potential to provide accessible and accurate health advice and triage recommendations to patients. If clinical safety is assured through reproducible evidence of diagnostic and triage accuracy, OSCs could have a valuable place in a sustainable health system, with the potential to support individuals to self-care more regularly for self-limiting conditions while also directing them to appropriate health care assessment when needed. This arrangement could also help to rationalize the use of health care products and services and reduce unnecessary pressure on HCPs and health systems in a variety of settings. Our review highlighted inconsistent evidence across the included studies regarding the triage and diagnostic accuracy of OSCs for general health concerns. As the congruent use of these tools continues to increase, especially after the advent of the COVID-19 pandemic, it is essential that researchers, developers, and HCPs work together and engage with users to ensure OSCs' safety and accuracy before their widescale adoption in home, community, and health care settings.

## Appendix

Multimedia Appendix 1 PRISMA (Preferred Reporting Items for Systematic Reviews and Meta-Analyses) checklist.

Multimedia Appendix 2 Search strategies.

Multimedia Appendix 3 Data extraction form with all extracted data.

Multimedia Appendix 4 List of online symptom checkers tested and measurements used by the included studies.

## Abbreviations

AI: artificial intelligence
CE: Conformité Européenne
ED: emergency department
GP: general practitioner
HCP: health care professional
HMIC: Health Management Information Consortium
LGBTQ: lesbian, gay, bisexual, transgender, and queer
MHRA: Medicines & Healthcare Products Regulatory Agency
NHS: National Health Service
NICE: National Institute for Health and Care Excellence
OSC: online symptom checker
PRISMA: Preferred Reporting Items for Systematic Reviews and Meta-Analyses
QUADAS-2: Quality Assessment of Diagnostic Accuracy Studies-2
UKCA: UK Conformity Assessed
WHO: World Health Organization

## Data Availability

The search strategies, the list of online symptom checkers tested and measurements used by the included studies, and data extraction table (including the data extracted from the included studies) are provided as multimedia appendices. Any desired additional data can be made available by authors upon request.

## References

[1] (2009). Health workforce: the health workforce crisis. World Health Organization.

[2] Lupton D, Jutel A (2015). 'It's like having a physician in your pocket!' A critical analysis of self-diagnosis smartphone apps. Soc Sci Med.

[3] Alwashmi MF (2020). The use of digital health in the detection and management of COVID-19. Int J Environ Res Public Health.

[4] North F, Ward WJ, Varkey P, Tulledge-Scheitel SM (2012). Should you search the internet for information about your acute symptom?. Telemed J E Health.

[5] Powley L, McIlroy G, Simons G, Raza K (2016). Are online symptoms checkers useful for patients with inflammatory arthritis?. BMC Musculoskelet Disord.

[6] Turner J, Knowles E, Simpson R, Sampson F, Dixon S, Long J, Bell-Gorrod H, Jacques R, Coster J, Yang H, Nicholl J, Bath P, Fall D (2021). Impact of NHS 111 online on the NHS 111 telephone service and urgent care system: a mixed-methods study. Health Serv Deliv Res.

[7] Aboueid S, Meyer S, Wallace JR, Mahajan S, Chaurasia A (2021). Young adults' perspectives on the use of symptom checkers for self-triage and self-diagnosis: qualitative study. JMIR Public Health Surveill.

[8] Meyer AN, Giardina TD, Spitzmueller C, Shahid U, Scott TM, Singh H (2020). Patient perspectives on the usefulness of an artificial intelligence-assisted symptom checker: cross-sectional survey study. J Med Internet Res.

[9] Liu AW, Odisho AY, Brown Iii W, Gonzales R, Neinstein AB, Judson TJ (2022). Patient experience and feedback after using an electronic health record-integrated COVID-19 symptom checker: survey study. JMIR Hum Factors.

[10] Perlman A, Vodonos Zilberg A, Bak P, Dreyfuss M, Leventer-Roberts M, Vurembrand Y, Jeffries HE, Fisher E, Steuerman Y, Namir Y, Goldschmidt Y, Souroujon D (2020). Characteristics and symptoms of app users seeking COVID-19-related digital health information and remote services: retrospective cohort study. J Med Internet Res.

[11] Wallace W, Chan C, Chidambaram S, Hanna L, Iqbal FM, Acharya A, Normahani P, Ashrafian H, Markar SR, Sounderajah V, Darzi A (2022). The diagnostic and triage accuracy of digital and online symptom checker tools: a systematic review. NPJ Digit Med.

[12] Chambers D, Cantrell AJ, Johnson M, Preston L, Baxter SK, Booth A, Turner J (2019). Digital and online symptom checkers and health assessment/triage services for urgent health problems: systematic review. BMJ Open.

[13] Page MJ, McKenzie JE, Bossuyt PM, Boutron I, Hoffmann TC, Mulrow CD, Shamseer L, Tetzlaff JM, Akl EA, Brennan SE, Chou R, Glanville J, Grimshaw JM, Hróbjartsson A, Lalu MM, Li T, Loder EW, Mayo-Wilson E, McDonald S, McGuinness LA, Stewart LA, Thomas J, Tricco AC, Welch VA, Whiting P, Moher D (2021). The PRISMA 2020 statement: an updated guideline for reporting systematic reviews. BMJ.

[14] Semigran HL, Linder JA, Gidengil C, Mehrotra A (2015). Evaluation of symptom checkers for self diagnosis and triage: audit study. BMJ.

[15] Semigran HL, Levine DM, Nundy S, Mehrotra A (2016). Comparison of physician and computer diagnostic accuracy. JAMA Intern Med.

[16] Berry AC, Cash BD, Wang B, Mulekar MS, Van Haneghan AB, Yuquimpo K, Swaney A, Marshall MC, Green WK (2019). Online symptom checker diagnostic and triage accuracy for HIV and hepatitis C. Epidemiol Infect.

[17] Gilbert S, Mehl A, Baluch A, Cawley C, Challiner J, Fraser H, Millen E, Montazeri M, Multmeier J, Pick F, Richter C, Türk E, Upadhyay S, Virani V, Vona N, Wicks P, Novorol C (2020). How accurate are digital symptom assessment apps for suggesting conditions and urgency advice? A clinical vignettes comparison to GPs. BMJ Open.

[18] Delshad S, Dontaraju VS, Chengat V (2021). Artificial intelligence-based application provides accurate medical triage advice when compared to consensus decisions of healthcare providers. Cureus.

[19] Poote AE, French DP, Dale J, Powell J (2014). A study of automated self-assessment in a primary care student health centre setting. J Telemed Telecare.

[20] Dickson SJ, Dewar C, Richardson A, Hunter A, Searle S, Hodgson LE (2022). Agreement and validity of electronic patient self-triage (eTriage) with nurse triage in two UK emergency departments: a retrospective study. Eur J Emerg Med.

[21] Ceney A, Tolond S, Glowinski A, Marks B, Swift S, Palser T (2021). Accuracy of online symptom checkers and the potential impact on service utilisation. PLoS One.

[22] Hill MG, Sim M, Mills B (2020). The quality of diagnosis and triage advice provided by free online symptom checkers and apps in Australia. Med J Aust.

[23] Gilbert S, Fenech M, Upadhyay S, Wicks P, Novorol C (2021). Quality of condition suggestions and urgency advice provided by the Ada symptom assessment app evaluated with vignettes optimised for Australia. Aust J Prim Health.

[24] Trivedi S, Littmann J, Stempien J, Kapur P, Bryce R, Betz M (2021). A comparison between computer-assisted self-triage by patients and triage performed by nurses in the emergency department. Cureus.

[25] Chan F, Lai S, Pieterman M, Richardson L, Singh A, Peters J, Toy A, Piccininni C, Rouault T, Wong K, Quong JK, Wakabayashi AT, Pawelec-Brzychczy A (2021). Performance of a new symptom checker in patient triage: Canadian cohort study. PLoS One.

[26] Verzantvoort NC, Teunis T, Verheij TJ, van der Velden AW (2018). Self-triage for acute primary care via a smartphone application: practical, safe and efficient?. PLoS One.

[27] Yu SW, Ma A, Tsang VH, Chung LS, Leung SC, Leung LP (2020). Triage accuracy of online symptom checkers for accident and emergency department patients. Hong Kong J Emerg Med.

[28] Whiting PF, Rutjes AW, Westwood ME, Mallett S, Deeks JJ, Reitsma JB, Leeflang MM, Sterne JA, Bossuyt PM, QUADAS-2 Group (2011). QUADAS-2: a revised tool for the quality assessment of diagnostic accuracy studies. Ann Intern Med.

[29] Kopka M, Feufel MA, Balzer F, Schmieding ML (2022). The triage capability of laypersons: retrospective exploratory analysis. JMIR Form Res.

[30] Mills B, Hill M, Buck J, Walter E, Howard K, Raisinger A, Smith E (2019). What constitutes an emergency ambulance call?. Australas J Paramedicine.

[31] Schmieding ML, Mörgeli R, Schmieding MA, Feufel MA, Balzer F (2021). Benchmarking triage capability of symptom checkers against that of medical laypersons: survey study. J Med Internet Res.

[32] O'Keeffe C, Mason S, Jacques R, Nicholl J (2018). Characterising non-urgent users of the emergency department (ED): a retrospective analysis of routine ED data. PLoS One.

[33] Arellano Carmona K, Chittamuru D, Kravitz RL, Ramondt S, Ramírez AS (2022). Health information seeking from an intelligent web-based symptom checker: cross-sectional questionnaire study. J Med Internet Res.

[34] Kopka M, Schmieding ML, Rieger T, Roesler E, Balzer F, Feufel MA (2022). Determinants of laypersons' trust in medical decision aids: randomized controlled trial. JMIR Hum Factors.

[35] Miller S, Gilbert S, Virani V, Wicks P (2020). Patients' utilization and perception of an artificial intelligence-based symptom assessment and advice technology in a British primary care waiting room: exploratory pilot study. JMIR Hum Factors.

[36] Lewis J, Stone T, Simpson R, Jacques R, O'Keeffe C, Croft S, Mason S (2021). Patient compliance with NHS 111 advice: analysis of adult call and ED attendance data 2013-2017. PLoS One.

[37] Painter A, Hayhoe B, Riboli-Sasco E, El-Osta A (2022). Online symptom checkers: recommendations for a vignette-based clinical evaluation standard. J Med Internet Res.

[38] Adebowale V, Rao M (2020). It’s time to act on racism in the NHS. BMJ.

[39] Williams DR, Mohammed SA (2013). Racism and health I: pathways and scientific evidence. Am Behav Sci.

[40] Bécares L, Kapadia D, Nazroo J (2020). Neglect of older ethnic minority people in UK research and policy. BMJ.

[41] Salway S, Holman D, Lee C, McGowan V, Ben-Shlomo Y, Saxena S, Nazroo J (2020). Transforming the health system for the UK's multiethnic population. BMJ.

[42] McInroy LB, McCloskey RJ, Craig SL, Eaton AD (2019). LGBTQ+ youths’ community engagement and resource seeking online versus offline. J Technol Hum Serv.

[43] Noor P (2020). Can we trust AI not to further embed racial bias and prejudice?. BMJ.

[44] Figueroa CA, Luo T, Aguilera A, Lyles CR (2021). The need for feminist intersectionality in digital health. Lancet Digit Health.

[45] Marco-Ruiz L, Bønes E, de la Asunción E, Gabarron E, Aviles-Solis JC, Lee E, Traver V, Sato K, Bellika JG (2017). Combining multivariate statistics and the think-aloud protocol to assess human-computer interaction barriers in symptom checkers. J Biomed Inform.

[46] Fraser H, Coiera E, Wong D (2018). Safety of patient-facing digital symptom checkers. Lancet.

[47] Talmon J, Ammenwerth E, Brender J, de Keizer N, Nykänen P, Rigby M (2009). STARE-HI--statement on reporting of evaluation studies in Health Informatics. Int J Med Inform.

[48] Stead WW, Haynes RB, Fuller S, Friedman CP, Travis LE, Beck JR, Fenichel CH, Chandrasekaran B, Buchanan BG, Abola EE, Sievert MC, Gardner RM, Jaffe JM, Pearson WP, Abarbanel RM (1994). Designing medical informatics research and library--resource projects to increase what is learned. J Am Med Inform Assoc.

[49] Murray E, Hekler EB, Andersson G, Collins LM, Doherty A, Hollis C, Rivera DE, West R, Wyatt JC (2016). Evaluating digital health interventions: key questions and approaches. Am J Prev Med.

[50] Millenson ML, Baldwin JL, Zipperer L, Singh H (2018). Beyond Dr. Google: the evidence on consumer-facing digital tools for diagnosis. Diagnosis (Berl).

[51] Jutel A, Lupton D (2015). Digitizing diagnosis: a review of mobile applications in the diagnostic process. Diagnosis (Berl).

