# Supplementary material for: Triage and Diagnostic Accuracy of Online Symptom Checkers: Systematic Review
Source: J Med Internet Res. 2023 Jun 2;25:e43803. doi: 10.2196/43803 (PMC10276326; doi:10.2196/43803)
Supplement: Multimedia Appendix 2 [file jmir_v25i1e43803_app2.doc]

**Multimedia Appendix 2: Search strategies**

**EMBASE**

1/ Self evaluation/

2/ (self triage$ or self diagnos?s or self refer$ or self assess$).mp

3/ (symptom$ adj2 check$).mp

4/ 1 OR 2 OR 3

5/ internet/

6/ (online or on-line or web or automated or internet or digital or app or apps or application$ or mobile$ or smartphone$ or smart phone$ or computer$ or remote or interactive).mp

7/ artificial intelligence or exp Artificial intelligence/ 46836

8/ 5 OR 6 OR 7

9/ triag* adj3 (web or internet or digital or automated or remote or electronic or mobile or app or apps or application* or smart phone* or smartphone* or device or interactive).mp

10/ (chatbot$ and (assess$ or triage$ or diagnos$)).mp

11/ 4 AND 8

13/ 11 OR 9 OR 10

**Ovid MEDLINE(R) Epub Ahead of Print, In-Process & Other Non-Indexed Citations, Ovid MEDLINE(R) Daily and Ovid MEDLINE(R)**

1 Diagnostic self evaluation/

2 (self triage$ or self diagnos?s or self refer$ or self assess$).mp

3 (symptom$ adj2 check$).mp

4 1 OR 2 OR 3

5 internet/

6 (online or on-line or web or automated or internet or digital or app or apps or application$ or mobile$ or smartphone$ or smart phone$ or computer$ or remote or interactive).mp

7 artificial intelligence or exp Artificial intelligence/

8 5 OR 6 OR 7

9 triag* adj3 (web or internet or digital or tool or automated or remote or electronic or mobile or app or apps or application* or smart phone* or smartphone* or device or interactive)).mp.

10 (chatbot$ and (assess$ or triage$ or diagnos$)).mp

11 4 AND 8

12 11 OR 9 OR 10

**Ovid HMIC**

1 self evaluation/

2 (self triage$ or self diagnos?s or self refer$ or self assess$).mp

3 (symptom$ adj2 check$).mp

4 1 OR 2 OR 3

5 internet/

6 (online or on-line or web or automated or internet or digital or app or apps or application$ or mobile$ or smartphone$ or smart phone$ or computer$ or remote or interactive or chatbot$).mp

7 artificial intelligence or exp Artificial intelligence/

8 5 OR 6 OR 7

9 (triag* adj3 (web or internet or digital or tool or checker or automated or remote or electronic or mobile or app or apps or application or smart phone* or smartphone* or device or interactive or chatbot$).mp

10 (chatbot$ and (assess$ or triage$ or diagnos$)).mp

11 4 AND 8

13 11 OR 9

**CINHAL (Date: 2010-2022; English Language; Peer Reviewed)**

S1 "self triage*" or "self diagnos?s" or "self refer*" or "self assess*"

S2 symptom$ N2 check*

S3 (MH "Internet") OR (MH "Internet-Based Intervention") OR (MH "World Wide Web Applications") OR (MH "Telepsychiatry") OR (MH "World Wide Web")

S4 (online or on-line or web or automated or internet or digital or app or apps or application$ or mobile$ or smartphone$ or smart phone$ or computer$ or remote or interactive or chatbot$)

S5 (MH "Artificial Intelligence")

S6 S3 OR S4 OR S5

S7 (MH "Self Diagnosis") OR (MH "Self Assessment")

S8 S1 OR S2 OR S7

S9 S6 AND S8

S10 chatbot$ AND ( assess$ or triage$ or diagnos$ )

S11 triag* N3 (web or internet or digital or automated or remote or electronic or mobile or app or apps or application or smart phone* or smartphone* or device or interactive)

S12 S9 OR S10 OR S11

**Web of Science Core Collection**

**2010 to 2022, English**, including Science Citation Index Expanded (SCI-EXPANDED), Social Sciences Citation Index (SSCI), Arts & Humanities Citation Index (A&HCI) and Emerging Sources Citation Index (ESCI);

**excluding** Conference Proceedings Citation Index- Science (CPCI-S) and Conference Proceedings Citation Index- Social Science & Humanities (CPCI-SSH)

#1 TS=("self triage*" or "self diagnos?s" or "self refer*" or "self assess*"

#2 TS=(symptom$ NEAR/2 check*)

#3 TS=(on line or web or automated or internet or digital or app$ or application$ or mobile$ or smartphone$ or smart phone$ or computer$ or remote or interactive or AI or artificial intelligence)

#4 (#2 OR #1)

#5 (#4 AND #3)

#6 TS=(chatbot$ and (assess* or triage$ or diagnos*))

#7 TS= ((web or internet or on$line or electronic or digital or remote or mobile or app$ or application$ or smart phone$ or smartphone$ or device or interactive) NEAR/3 triage)

**#8 (#5 or #6 or #7)**
