# Supplementary material for: Triage and Diagnostic Accuracy of Online Symptom Checkers: Systematic Review
Source: J Med Internet Res. 2023 Jun 2;25:e43803. doi: 10.2196/43803 (PMC10276326; doi:10.2196/43803)
Supplement: Multimedia Appendix 4 [file jmir_v25i1e43803_app4.doc]

**Multimedia Appendix 4:**

**List of OSCs tested & measurement used by included studies**

| **Reference** | **OSCs tested** | **Measurement of diagnostic accuracy** | **Measurement of triage accuracy** |
| --- | --- | --- | --- |
| Poote 2014 | Prototype adapted from a widely used telephone triage system that supports nurses' decision-making in primary care & has been evaluated in previous studies in the UK and Switzerland. The modification involved creating a new user interface that presented the content in an intuitive format suitable for online completion by lay people, with the questions & answers translated into nontechnical, plain English. |  | 1. Correct triage (agreement between the triage recommendation of the self-assessment system and the assessment of urgency made by the GP) 2. Under/over-triage  **Variables:**  a. Prototype version b. Age & gender c. Types of symptoms |
| Semigran 2015 | 1. AskMD (USA)  2. BetterMedicine (USA)  3. DocResponse (USA)  4. Doctor Diagnose (USA)  5. Drugs.com (USA)  6. EarlyDoc (Netherlands) - discontinued 7. Esagil (USA)  8. Family Doctor (USA)  9. FreeMD (USA) - discontinued 10. Harvard Medical School Family Health Guide (USA) - discontinued 11. Healthline (USA)  12. Healthwise (USA)  13. Healthy Children (USA)  14. Isabel (UK)  15. iTriage (USA)  16. Mayo Clinic (USA) 17. MEDoctor (USA)  18. NHS Symptom Checkers (UK) 19. Steps2Care (USA) - discontinued  20. Symcat (USA)  21. Symptify (USA) 22. Symptomate (Poland)  23. WebMD (USA) | **Ranking of correct diagnosis** 1. Listed 1^st^  2. Listed in top 3  3. Listed in top 20  **Variables:**  **Type of condition:** a/ severity/urgency; b/frequency **OSC's characteristics:** c/ demographic questions; type of OSC | 1. Correct triage 2. Under/over-triage **Variables:**  Type of condition: a. severity; b. frequency c. OSC's characteristics |
| Semigran 2016 | 1. AskMD (USA)  2. BetterMedicine (USA)  3. DocResponse (USA)  4. Doctor Diagnose (USA)  5. Drugs.com (USA)  6. EarlyDoc (Netherlands)  7. Esagil (USA)  8. Family Doctor (USA)  9. FreeMD (USA)  10. Harvard Medical School Family Health Guide (USA) 11. Healthline (USA)  12. Healthwise (USA)  13. Healthy Children (USA)  14. Isabel (UK)  15. iTriage (USA)  16. Mayo Clinic (USA) 17. MEDoctor (USA)  18. NHS Symptom Checkers (UK) 19. Steps2Care (USA)  20. Symcat (USA)  21. Symptify (USA) 22. Symptomate (Poland)  23. WebMD (USA) | **Ranking of correct diagnosis** 1. Listed 1^st^  2. Listed in top 3  **Variables:**  Type of condition: a/ severity/urgency; b/frequency OSC's characteristics: c/ demographic questions; type of OSC |  |
| Verzantvoort 2018 | App *Should I see a doctor?* developed as a self-triage decision support tool for acute primary care in the Netherlands, based on the Dutch Triage System (NTS) and the NHG guidelines |  | **1. Correct triage** (agreement between app and nurse) **2. Under/over-triage** |
| Berry 2019 | 1. Mayo Clinic 2. WebMD 3. Symptomate 4. Symcat 5. Isabel | **Ranking of correct diagnosis** 1. Listed 1^st^  2. Listed in top 3 3. Listed in top 10 4. Listed at all 5. Mean rank of correct diagnosis  **Variables:**  Initial diagnosis: HIV/Hep C/both OSC | Correct triage  Note: Since patients are in ED, expected to all be emergent |
| Gilbert 2020 | 1. Ada  2. Babylon  3. Buoy  4. K Health  5. Mediktor  6. Symptomate  7. Your.MD  8. WebMD | **Ranking of correct diagnosis** 1. Listed 1^st^  2. Listed in top 3 3. Listed in top 5  **Variables:**  a. OSC b. NHS vs non-NHS vignette c. required-answer vs provided-answer | 1. Safe VS unsafe (safe = max 1 level less conservative than gold-standard) 2. % of vignettes for which GPs & OSCs were: a/ over conservative; b/ overconservative but suitable (one level too high); c/ exactly-matched; d/ safe but underconservative (one level too low); e/ potentially unsafe. |
| Hill 2020 | 1. AARP Health Tools Symptoms 2. Alberta Check Your Symptoms (Healthwise) 3. APWU Health Plan Helpful tool: Symptom Checker (American Postal Workers Union Health Plan) 4. Buoy Symptom Checker 5. Children's Wisconsin Symptom Checker 6. Drugs.com Symptom Checker 7. Everyday Health Healthy Living Symptom Checker 8. Family Doctor Symptom Checker 9. Healthdirect Symptom Checker 10. Healthline Symptom Checker 11. HealthLinkBC Check Your Symptoms 12. Health Status Symptom Checker 13. Healthy Children.org KidsDoc Symptom Checker 14. Isabel 15. John Hopkins All Children's Hospital Symptom Checker 16. Mayo Clinic Symptom Checker 17. MedicineNet Symptom Checker: Symptoms & Signs A-Z 18. Michigan Medicine Symptom Checker (University of Michigan) 19. Patient Symptom Checker 20. Right Diagnosis from healthgrades Symptom Checker 21. RxList Symptom Checker powered by WebMD 22. St Luke's Symptom Checker 23. Symcat.com What is bothering you today? 24. Symptomate 25. WebMD Symptom Checker 26. What's My Diagnosis 27. Doctor Diagnose Symptoms Check (Google Play) 28. Drugs.com Medication Guide Symptom Checker (Google Play) 29. ePain Assist Symptom Checker (Apple App) [no longer available] 30. ePain Assist Symptom Checker (Google Play) 31. Healthdirect Australian health advice (Apple App) 32. Symptify (Google Play) no longer available 33. Symptomate Check your symptoms (Apple App) 34. Symptomate – Symptom checker (Google Play) 35. WebMD: Symptoms, Doctors, & Rx (Apple App) 36. WebMD: Check Symptoms, Find Doctors, & Rx Savings (Google Play) | **Ranking of correct diagnosis** 1. Listed 1^st^  2. Listed in top 3 3. Listed in top 10  **Variables:**  T**ype of condition:** a/ severity; b/frequency **OSC's characteristics:** c/ AI algorythms; d/ demographic questions; e/ max nb of diagnoses ; Google VS Apple | **Correct triage**  **Variables:**  **1. type of condition:** a/ severity; b/frequency **2. OSC's characteristics:** a/ AI algorythms; b/ demographic questions; c/ max nb of diagnoses |
| Yu 2020 | 1. Drugs.com 2. FamilyDoctor |  | **1. Correct triage 2. Under/over-triage 3. Sensitivity; specificity; PPV; NPV   Variables:  a. OSC**: Drugs.com VS FamilyDoctor  b. **Severity** (triage category) |
| Ceney 2021 | 1. Ada Health 2. Ask NHS Sensely 3. Babylon Health 4. Buoy Health 5. Clinova 6. Doctorlink 7. Infermedica 8. Isabel Health 9. Mediktor 10. NHS 111 11. Web.MD 12. Your.MD | **Ranking of correct diagnosis** 1. Listed 1st 2. Listed in top 5 3. Listed at all  **Variables:**  **Condition** a/ level of urgency **OSC's** b/ nb of questions c/ time to complete d/demographic questions | **1. Correct triage**  **2. Under/over-triage (safety of the triage)**  **Variables:  Condition a/** level of urgency **OSC's b/** nb of questions |
| Chan 2021 | A new (prototype) symptom checker designed by author FC and coded by computer science students at Western University |  | 1. Correct triage  2. Under/over-triage (safety of the triage) 3. Sensitivity  **Variables:** a/ patients recruited at ED b/ patients recruited in primary care setting |
| Delshad 2021 | MayaMD |  | Triage in agreement with individual & consensus clinical triage assessment in 3 phases |
| Gilbert 2021 | Ada | **Ranking of correct diagnosis** 1. Listed 1^st^  2. Listed in top 3 3. Listed in top 5  **Variable:**  General vs Australian specific vignettes | Correct Triage  **Variables:** 1. severity of condition 2. General vignettes vs specifically designed for Australian context |
| Trivedi 2021 | Algorithmic questionnaire (series of "Yes/No" answers displayed electronically in algorithmic sequence), transcribed using FluidSurvey (SurveyMonkey Canada Inc., Ottawa, Canada). The questionnaire was developed a priori by the authors without referral to previously published algorithms. Fundamental premise: short to be easily answered by patients with limited health literacy & directed toward the most common presenting complaints. It was completed by the patient or caregiver on the tablet and subjects were asked to predict whether they would require admission to the hospital. At the end of the questionnaire, the an algorithm-generated self-triage score (AGST) score was assigned, based on the answers provided. |  | Agreement between AGST: algorithm-generated self-triage score and CTAS: Canadian Triage and Acuity Scale  **Variables:** 1. age 2. sex 3. triage category 4. heart- or lung-related complaints |
| Dickson 2022 | eTriage, an automated digital check-in & triage solution developed in the UK by practicing NHS clinicians. |  | 1. Correct triage  2. Triage within 1 triage category 3 Triage within 2 triage categories 4. Triage within >2 triage categories  5. Under/over-triage (safety of the triage) **Variables:** Condition's emergency |
